# The effect of using fruit peel on broiler growth and health

**DOI:** 10.14202/vetworld.2023.987-1000

**Published:** 2023-05-13

**Authors:** Sugiharto Sugiharto

**Affiliations:** Department of Animal Science, Faculty of Animal and Agricultural Sciences, Universitas Diponegoro, Semarang, Central Java, Indonesia

**Keywords:** antibacterial, antioxidants, byproduct, feed, immune system

## Abstract

To ensure the long-term viability of broiler farming, producers must address a number of issues, including rising feed costs, a ban on antibiotic growth promoters, and growing consumer awareness of chemical residues in broiler chicken meat. Fruit peel is a waste with no commercial value, but due to its high nutritional content, particularly in terms of energy, it has the potential to be used as an alternative feed source for broiler chicks. Fruit peel also contains a number of nutraceutical compounds that have the potential to be added to feed or used as natural supplements for broiler chickens due to their antibacterial, antioxidant, and immunostimulant properties. Fruit peels have high fiber content and antinutritional and toxic components that may interfere with broiler digestion and physiological function, so they should be used cautiously in broiler production. Various processes, including fermentation, extraction, distillation, and combining with other active components, such as enzymes, may be used to optimize the use of fruit peels in broiler production. This review examines the use of fruit peel and its effects on broiler growth and health.

## Introduction

The growth and health of broilers are highly dependent on their diets, both in terms of quality and quantity. Broiler farmers are looking for alternative feed ingredients to reduce feed costs because broiler feed prices are constantly rising. Fruit peel is a waste with no economic value and potentially harming the environment if not properly handled [1, 2]. Indeed, several studies have confirmed that fruit peel has the potential to be used as a feed ingredient for broiler chickens due to its high-energy content [3–5]. For the record, energy constitutes the majority of constituents in broiler rations and the majority of expenditures. Therefore, using fruit peels as an alternative energy source feed ingredient can help to reduce broiler feed costs.

Following the ban on antibiotic growth promoters (AGPs) in feed, commercial broiler producers must act immediately to employ feed additives or supplements to preserve health and maximize growth potential [6–8]. To ensure the long-term viability of broiler farming, broiler producers should be concerned about the growing public awareness of the negative effects of chemical-based feed additives or supplements (such as synthetic antioxidants) on consumer health. According to studies [9–11], fruit peel contains a variety of active compounds that can improve the health of broiler chickens. Fruit peels typically contain antibacterial, antioxidant, immunomodulatory, and anti-inflammatory active components, all of which can improve broiler chicken health. Aside from its potential as an alternative feed ingredient or feed supplement, the use of fruit peel for broiler chickens must be done with caution, taking into account the content of crude fiber, antinutritional compounds, and toxins, the presence of which varies between fruit peels [10–13]. Fruit peels can be used more effectively as feed additives or alternative feed ingredients for broilers through various processes. The process includes fermentation, extraction, distillation, and pairing with other active ingredients, such as enzymes.

This review focuses on fruit peels’ productivity and health benefits in broiler production. This review also explains how fruit peels can affect the growth and well-being of chickens. A number of alternative approaches to optimizing the use of fruit peels as substitute feed ingredients and feed additives for broiler chickens are also discussed.

## Availability of Fruit Peels

The fruit peel protects the fruits by being the outermost layer. Fruit peel is a waste that, if not properly managed, can cause environmental problems [1, 2]. Peels are produced in large quantities while processing fruits for food and industrial purposes. The average peel weight is 15%–20% of the fruit weight, but during manual peeling, it can reach 25%–30% [12]. Romelle *et al*. [13] also reported the potential for fruit peel production from various fruits, with peels accounting for nearly 30% of total fruit weight. In particular, papaya produced 10.21-g peel/100-g fresh weight fruit, pineapple 9.17, mango 9.94, apple 10.20, banana 33.81, orange 14.27, pomegranate 11.69, and watermelon 6.44.

Global fruit production is increasing year after year. Shahbandeh [14] reported fruit production of 888.5 million metric tons in 2019, 899.56 million metric tons in 2020, and 909.64 million metric tons in 2021. This increase in fruit production will directly impact the production of fruit peels. Based on the information presented above, the use of fruit peels as feed ingredients or feed additives for broiler chickens has a high potential.

### The Nutrients and Active Components of Fruit Peels

Studies have shown that fruit peels are rich in nutrients (including dietary fiber, vitamins, and minerals), phytochemicals, antioxidants, and antifungal and antimicrobial compounds [2, 15], which are beneficial for poultry health. Dias *et al*. [2] recently collected several fruit peels from Sri Lanka’s food processing industries, including pineapple, orange, yellow passion fruit, and avocado. All fruit peels were found to be high in fiber and minerals. In particular, avocado peel and orange peel have a high-fat content. They can be used as a fat source in poultry feed as a substitute for animal fat sources. According to Nur *et al*. [16], the peel of *Pouteria campechiana* fruit contains 4.15% crude fiber, 7.46% fat, 41.54% carbohydrate, and 247.28 kcal/100 g energy. Nur *et al*. [16] further recommended that the peel of *P. campechiana* fruit is a good source of fat. Ribeiro *et al*. [17] revealed that the peel of umbu fruit (*Spondias tuberosa*) contains total solids 90.33 g/100 g dry weight, ash 3.38 g/100 g dry weight, protein 6.08 g/100 g dry weight, lipid 0.78 g/100 g dry weight, and fiber 49.34 g/100 g dry weight. Sadef *et al*. [18] showed that mango peel contains 3.5 ash, 0.34 lipid, 4.5 fiber, 4.30 protein, 78.5% dry matter carbohydrates, and 335 kcal/100 g energy. They also discovered that apple peel contains 2.6% dry matter of ash, 0.18% dry matter of lipid, 7.6% dry matter of fiber, 1.24% dry matter of protein, 76.5% dry matter carbohydrates, and 313 kcal/100 g energy. Other studies have also documented the proximate compositions of some fruit peels, including studies by Siyal *et al*. [19] on orange and banana peels, Ebrahimi *et al*. [20] on dried sweet orange (*Citrus sinensis*) peel, Akintunde *et al*. [11] on *Carica papaya* fruit peel, Otu *et al*. [21] on watermelon rind, and Vlaicu *et al*. [10] on orange and grapefruit peels. Romelle *et al*. [13] also reported the proximate composition of various fruit peels, including fruit peels of pawpaw, pineapple, mango, apple, banana, orange, pomegranate, and watermelon. Overall, the proximate composition of each fruit peel varies greatly depending on the fruit peel type, the fruit ripening stage, and the peeling method (manual peeling produces more fruit peels) [10–13].

Fruit peels have yielded a number of active components. In general, these active components exhibit antioxidant, antibacterial, antifungal, immunomodulatory, and anti-inflammatory properties (Table-1) [10, 11, 17, 22–28], which can contribute to the health and growth of broiler chickens. Indeed, each fruit peel contains specific active components and nutraceutical and functional properties. The antioxidant component is the most dominant active ingredient in fruit peels. In this case, the presence of phenolic components in fruit peels greatly contributes to the potential of fruit peels as an antioxidant source [9]. In addition, trace minerals, such as zinc and magnesium, are crucial for fruit peel’s capacity as a source of natural antioxidants [10, 11]. Another component that contributes significantly to the antioxidant capacity of fruit peels is condensed tannin [22]. Similarly, the pigments and vitamins (particularly C and E) found in fruit peels can significantly contribute to the antioxidant activity of fruit peels [11, 17]. Fruit peel has great potential as an antibacterial agent, as evidenced by the ability of avocado and coconut peel extracts to inhibit the growth of *Staphylococcus aureus*, *Shigella dysenteriae*, and *Candida albicans* bacteria in the study of Aguilar-Méndez *et al*. [9]. Several components of fruit peels, including the phenolic components, have been reported to contribute to antibacterial activity [9]. According to Juhari *et al*. [29], the antimicrobial components in fruit peels are typically secondary metabolites, such as phenolic compounds, steroids, and alkaloids. All these substances found in fruit peels inhibit the growth of pathogenic microorganisms [29]. In line with the previous study, Saleem and Saeed [30] reported that the high phenols, zinc, and magnesium content in yellow lemon peel contributed significantly to its antibacterial capacity. casein this case, surface area and surface defects of zinc and magnesium metal oxide particles play an important role in antimicrobial activities.

**Table-1:** Functional activities and bioactive compounds in some selected fruit peels.

Fruit peels	Functional activities	Active compounds in peels	Levels	Reference
Orange peel	Antioxidant, antibacterial and immunomodulatory potential	Vitamin E	100.50 mg/kg	[10]
		Lutein and zeaxanthin	81.52 mg/kg	
		Polyphenols	8.035 mg/g	
		Zinc	4.74 mg/kg	
		Antioxidant capacity	238.51 mmoli/kg equiv. ascorbic acid	
		Antioxidant capacity	231.33 mmoli/kg equiv. vitamin E	
Grapefruit peel	Antioxidant and antibacterial potential	Vitamin E	89.93 mg/kg	[10]
		Lutein and zeaxanthin	36.46 mg/kg	
		Polyphenols	12.162 mg/g	
		Zinc	5.41 mg/kg	
		Antioxidant capacity	238.25 mmoli/kg equiv. ascorbic acid	
		Antioxidant capacity	227.75 mmoli/kg equiv. vitamin E	
*Carica papaya* peel meal	Antioxidant and antibacterial potential	Saponin	9.69 mg/100 g	[11]
		Alkaloid	6.44 mg/100 g	
		Tannin	76.89 mg/100 g	
		Vitamin A	1795 IU/kg	
		Vitamin C	6.69 mg/100 g	
Umbu peel meal	Antioxidant and antibacterial potential	Lutein	54 µg/100 g DM	[17]
		Zeaxanthin	24 µg/100 g DM	
		Zeinoxanthin	441 µg/100 g DM	
		β-cryptoxanthin	420 µg/100 g DM	
		α-carotene	48 µg/100 g DM	
		β-carotene	1,276 µg/100 g DM	
		13-cis-β-carotene	60 µg/100 g DM	
		9-cis-β-carotene	181 µg/100 g DM	
Pomegranate peel	Antioxidant, antibacterial and immunomodulatory potential	Total polyphenol	179.21 mg gallic acid equiv./g DM	[22]
		Hydrolysable tannins	203.12 mg tannic acid/g DM	
		Condensed tannins	6.05 mg cyaniding acid/g DM	
Pomegranate peel	Antioxidant, antibacterial and immunomodulatory potential	Total phenolic compounds	331 mg/g	[23]
		Proanthocyanin	33.6 mg/g	
		Punicalagin	26.3 mg/g	
		Ellagic acid	1.12 mg/g	
		Oleuropein	2.18 mg/g	
		Gallic acid	3.38 mg/g	
		Caffeic acid	0.93 mg/g	
		Catechin	0.63 mg/g	
Banana peel	Antioxidant antibacterial, and immunomodulatory potential	Phenols	872.7 mg/100 g	[24]
		Gallocatechin	91.9 mg/100 g	
		Epigallocatechin	65.9 mg/100 g	
		Epigallocatechin gallate	10.3 mg/100 g	
Pomegranate peel extract	Antioxidant, antibacterial and immunomodulatory potential	Polyphenols	143.98 mg GAE/g	[25]
		Flavonols	16.75 mg QE/g	
Sweet orange (*Citrus sinensis*) peel	Antioxidant and anti-inflammatory potential	Vitamin C	49 IU/kg	[26]
Sweet orange (*Citrus sinensis*) peel	Antioxidant, antibacterial, antifungal and anti-inflammatory potentials	Retinol	85.71 IU	[27]
		Ascorbic acid	12.91 mg/100 g	
		Niacin	0.81 mg/100 g	
		Riboflavin	0.15 mg/100 g	
		Thiamine	0.11 mg/100 g	
Guavira fruit (*Campomanesia adamantium*) peel extract	Antioxidant and anti-inflammatory potentials	Polyphenols	47.80 mg GAE/L	[28]
		Flavonoids	10.99 mgQE/L	

DM=Dry matter, GAE=Gallic acid equivalents, QE=Quercetin equivalent

Studies show that fruit peels have the potential to act as immunomodulators/immunostimulators and anti-inflammatory agents (Table-1). These two fruit peel characteristics are important for broiler chickens’ ability to respond to invading pathogens. Because anthocyanins can mimic pathogen-related molecular patterns and make γδT cells of the immune system work in a proactive mode, the immunostimulatory capacity of fruit peels is often associated with their content of anthocyanins, which can trigger immune cells [31]. Furthermore, polyphenols are bioactive compounds found in many fruit peels that can improve gut health by regulating immunity and intestinal mucosa inflammation [8, 31]. Polyphenols can increase the number of intraepithelial T-cells and mucosal eosinophils in the context of polyphenols enhancing intestinal mucosal immunity. Polyphenols, such as epigallocatechin-3-gallate, epicatechin-3-gallate, or epigallocatechin, have also been shown to increase white blood cell (WBC) production of interleukin-10 (IL-10). Therefore, polyphenols are responsible for decreasing the activity of pro-inflammatory cytokines secreted by macrophages while increasing the activity of anti-inflammatory cytokines [31]. Antioxidant vitamins (i.e., Vitamins C and E) have also been linked to fruit peels’ immunomodulatory or immunostimulatory effects. These vitamins can protect lymphoid organs and tissues from the harmful activity of pro-oxidants or free radicals (i.e., vitamins can scavenge free radicals), which have the potential to damage the lymphoid organs or tissues as well as immune cell maturation sites in the body [31, 32]. Apart from its antinutritional properties, tannin is an active ingredient in fruit peels with antioxidant properties and the ability to stimulate phagocytic cells. In addition, tannins have been shown to have the ability in anti-inflammatory and immunomodulatory activities [31, 33]. Malleshappa *et al*. [34] reported that the anti-inflammatory effect elicited by fruit peels can be associated individually or collectively with flavonoids, limonoids, steroids, alkaloids, terpenes, and tannins. The types and levels of active ingredients found in fruit peels, like their proximate composition, vary widely. Saleem and Saeed [30] reported that differences in genotypes and fruit type significantly impact the type and level of active components in fruit peels. It has also been reported that the ripening stage influences the content of active components in fruit peels [11]. Differences in solvents used during extraction can also affect the amount of active ingredients that can be produced from fruit peels in the case of extracts [30].

Although fruit peels have a high potential for use as a source of nutraceutical components for the health and productivity of broiler chickens, studies have reported a number of antinutritive components and toxins in fruit peels. The presence of these antinutrients and toxins can have a negative impact on health and broiler chicken growth. Romelle *et al*. [13] reported antinutritional and toxin components in pawpaw, pineapple, mango, apple, banana, orange, pomegranate, and watermelon peels. These anti-nutritional components include oxalates, hydrogen cyanides, alkaloids, and phytates. In line with this, Ani and Abel [32] reported several antinutritional substances in *Citrus maxima* peel extract, including phytic acid, tannin, and oxalate, with phytic acid being the most antinutritional substance among the three antinutrients. Several antinutritional substances and toxins are also found in several varieties of citrus fruits, including tannins, saponins, phytate, oxalate, flavonoids, and limonene. In general, the antinutrient content of fruits varies depending on the type [13]. Similarly, the antinutrient and toxin content of fruit peels may be affected by fruit variety [35].

## Fruit Peels as Alternative Feed Ingredients and Feed Additives for Broilers

In the midst of increasing broiler production costs, using waste or byproducts from the food industry as an alternative feed ingredient or feed additive may be a viable option. Fruit peels are a by-product of the food industry. Fruit peels are a potential alternative feed ingredient and feed additive for broilers due to their nutritional content and various active ingredients, as described above. However, in addition to having a low protein content in general, the use of fruit peels as a poultry feed ingredient is limited due to the presence of cyanogenic and phytate components, as well as rapid decay if not processed immediately [5, 12]. For the record, fruit peel is an organic material with a high-water content, so if it is not dried immediately, it can become a medium for developing decomposer microbes, which can harm the nutrient content and active ingredients in the fruit peel [2]. Another factor to consider when using fruit peel in broiler feed is its high fiber content, which can reduce nutrient digestibility in broiler chickens [22].

In general, there are two types of uses for fruit peel in broiler feed. The first category includes fruit peel, which can be used as an alternative feed ingredient to reduce the proportion of energy source feed ingredients in broiler rations. Previously, Agu *et al*. [3] used sweet orange (*C. sinensis*) fruit peel to substitute maize in broiler starter and finisher feeds at levels of 10%, 20%, 30%, 40%, and 50%. Fruit meal is typically fed to broiler chickens in the form of powder, which is made by crushing fruit peels after drying. The use of fruit peel meal as an alternative feed ingredient is expected to lower feed costs, which have recently increased in price [5]. Aside from being used as an alternative feed ingredient to replace energy sources, fruit peel meal as a feed ingredient in broiler rations is often used as a functional feed ingredient [36]. This is related to the presence of various active ingredients in fruit peel meal, such as antimicrobial, antioxidant, anti-inflammatory, immunomodulatory, and other components. In his recent review paper, Diarra [12] discussed the use of various types of peel meal as an alternative feed ingredient for broiler chickens in great detail. The second type of use of fruit peel is as a feed additive or feed supplement given to broiler chickens as an alternative to AGP or synthetic antioxidants [23, 37]. Fruit peels are typically fed to broiler chickens in the form of extract as feed additives and feed supplements. In this context, extraction can release the active ingredients in the fruit peel from complex glycoside bonds, increasing the active components, and making it easier to use broilers. Furthermore, the extraction process can potentially reduce the antinutritional components in the fruit peels.

## Effect of Fruit Peels on Growth Performance of Broilers

Growth performance and feed efficiency are important parameters for broiler producers because they are related to feed costs, which continue to increase. Farmers must use feed additives selectively in the current post-antibiotic (AGP) era to maximize the genetic potential for broiler growth [38]. Many AGP alternatives are currently available to broiler farmers, including probiotics, prebiotics, enzymes, synbiotics, and organic acids. However, the high cost of procuring feed additives is often an additional burden for broiler farmers already burdened by the high feed cost. Functional feed ingredients may be able to address the high cost of commercially available feed additives. According to Sugiharto *et al*. [36], functional feed ingredients can be a source of energy or protein and a growth promoter, antibacterial, antioxidant, immunomodulator, and other functions. In this case, using fruit peel as a functional feed ingredient can address two problems at once, namely, the high cost of conventional feed as a source of protein or energy while also acting as a substitute for AGP and antioxidants [36].

Table-2 shows several studies that used fruit peels as alternative feed ingredients and feed additives for broiler chickens [5, 19–21, 23, 28, 37, 39–47]. The relatively high carbohydrate, fat, and energy contents of fruit peel meal are factors that poultry nutritionists consider when using fruit peel as an alternative feed ingredient, particularly to reduce conventional energy sources [5, 39]. Adekeye *et al*. [5] reduce the proportion of energy source feed ingredients (corn and wheat bran) in broiler rations using cassava peel meal. They reported that using as much as 150 kg/t of cassava peel meal can improve growth performance while saving money. In another study, Badr *et al*. [39] used prickly pear fruit peel powder to reduce the proportion of yellow corn. They showed that prickly pear fruit peel powder improved growth rate, feed conversion ratio (FCR), net protein utilization, carcass traits, physical and chemical measurements of broiler meat, and biological value of meat at all levels (5%, 10%, and 15% of diets).

**Table-2:** Effects of fruit peels on growth performance of broilers.

Fruit peels	Active compounds	Levels in diets	Effects on broilers	Reference
Cassava peel meal	NM	150–300 kg/t feed	Inclusion at 150 kg/t improved growth performance and save cost	[5]
Orange peel or banana peel meal	NM	1.5% and 3.0%	At both levels increased final body weight, carcass weight and dressing percentage of broiler	[19]
Sweet orange peel meal	Vitamin C and polyphenols	1.5% and 3.0%	At 1.5% promoted feed intake and weight gain during 1–21 days of age	[20]
Dried watermelon rind	NM	0, 25, 50, 75 and 100% in replacement of wheat offal	The replacement of wheat offal at 25% improved crude fiber digestibility and total digestible nutrient for broilers at starter phase	[21]
Pomegranate peel extract	Total phenolic compounds, proanthocyanin, punicalagin, ellagic acid, oleuropein, gallic acid, caffeic acid and catechin	2.5, 5.0, 7.5 and 10 g/kg	At 7.5 g/kg improved FCR and at 7.5 and 10 g/kg reduced abdominal fat content of broilers	[23]
Guavira fruit (*Campomanesia adamantium*) peel extract	Phenols and flavonoids	100–500 mg/kg	At the levels of 200–500 mg/kg increased weight gain of broilers	[28]
Pomegranate peel extract	Phenolic compounds and flavonoids	0.05 and 0.10%	At 0.05% improved final body weight, body weight gain and FCR of broilers	[37]
Prickly pear fruits peel powder	Phenols and flavonoid	5, 10 and 15% of diets	At all levels improved growth rate, FCR, net protein utilization carcass traits, meat quality (physical and chemical), and biological value of meat	[39]
Pomegranate peel powder	Polyphenols and flavonols	2, 4, 6 and 8 g/kg feed	At 2 and 4 g/kg improved growth, FCR, nutrient digestibility and protein efficiency of broilers	[40]
Pomegranate peel infusion	Polyphenols	50 mL/L drinking water	Improved final body weight, daily body weight gain, and FCR of broilers	[41]
Red dragon fruit peel meal	NM	0.5, 1.0 and 1.5% of diets	Enhanced growth rate and carcass percentage, while decreasing abdominal fat pad of broilers	[42]
Pomegranate peel powder	Phenols	2, 3 and 4 g/kg diets	Improved growth rate and meat quality of broilers	[43]
Pomegranate peel powder	Flavonoids, catechins, ellagic acid, flavones, and anthocyanidins	0.25, 0.50, 1.00, and 1.50%	Increased growth rate and carcass percentage, reduced FCR and abdominal fat content of broilers	[44]
Pomegranate peel extract	Phenolic compounds, flavonoids, saponins and tannins	10 g/100 kg diets	Improved growth rate, feed intake, FCR and carcass traits of broilers	[45]
Sweet citrus peel powder	NM	2, 4, and 6 g/L of water	Increased weight gain and decreased FCR of broiler	[46]
Orange peel and lemon peel meal	NM	0.5% and 1.0%	Improved growth, FCR and dressing percentage of broilers	[47]

FCR=Feed conversion ratio, NM=Not mentioned

There are currently few studies that examine the cost savings associated with using fruit peel as a feed ingredient in broiler rations. However, adding fruit peels to the ration can typically save feed expenses, particularly when purchasing protein and energy feed sources [4, 5]. Fruit peels have a low economic value, but when used as a functional feed ingredient, they can improve bird health and growth [39], increasing feed efficiency and lowering illness treatment costs.

The improved growth performance in broiler chickens fed fruit peels is often attributed to the biological activity of various active components found in fruit peels. The antimicrobial components in fruit peels can improve the health and function of the digestive tract, thereby increasing nutrient digestibility, protein efficiency, and broiler chicken growth [21, 40]. Moreover, dietary supplementation with fruit peel improved broiler metabolic rate and, thus growth rate. According to Akbarian *et al*. [48], dietary administration of lemon peel extract increased 3,5,3′-triiodothyronine and growth hormone concentrations, resulting in an increased broiler growth rate. Fruit peels have also been shown to improve antioxidant status in broiler chickens [23, 24]. Ahmadipour *et al*. [23] reported that using pomegranate peel extract in feed can increase antioxidant enzyme (catalase [CAT] and superoxide dismutase [SOD]) activities and prevent lipid peroxidation in broiler chickens. According to Chueh *et al*. [24], using banana peel powder in feed can improve the antioxidant status of broiler chickens, as evidenced by increased SOD activity and decreased serum malondialdehyde (MDA) levels. Indicators of improved antioxidant status, such as SOD, CAT, and glutathione peroxidase (GPx), increased in response to banana peel powder administration. In general, improving antioxidant status can help to prevent potential oxidative damage that can interfere with physiological activities in broiler chickens. Furthermore, improving antioxidant status can reduce energy allocation for maintenance, allocating most of the energy to chicken growth.

Improving the immune system in chickens is often associated with increased growth rates in broiler chickens fed fruit peels. Pourhossein *et al*. [49] reported that feeding dried sweet orange (*C. sinensis*) peel to broiler chickens can improve their immune systems, as evidenced by an increase in total anti-sheep red blood cell (RBC) titer, immunoglobulin (Ig)G, and IgM levels. Pomegranate peel extract was also shown to increase serum lysozyme activity and the relative weight of the Fabricius bursa and thymus gland in broiler chickens [37]. Improving the immune response improves health status and thus reduces the allocation of energy for recovery, allowing more energy to be allocated for growth.

Not only does the use of fruit peels improve the growth rate and efficiency of feed use but it is also said to improve the carcass and meat characteristics of broiler chickens. Siyal *et al*. [19] reported that, besides increasing final body weight, including orange and banana peel meals in the ration can increase carcass weight and dressing percentage in broiler chickens. In agreement, Wijana *et al*. [50] revealed that drinking water administration of dragon fruit (*Hylocereus* spp.) peel extract increased the carcass percentage of chickens. Furthermore, Akuru *et al*. [25] reported that pomegranate peel powder meal administration in diets significantly improved broiler meat characteristics, such as increased water binding capacity, decreased cooking loss, and improved the antioxidant status of broiler meat. Vlaicu *et al*. [10] found that orange and grapefruit peels can improve the quality of broiler chicken meat by inhibiting fat peroxidation in the meat. The increase in carcass weight and dressing percentage is most likely related to increased nutrient digestibility, particularly protein, which results in increased protein accretion and, thus an increase in broiler chicken dressing percentage [50]. Furthermore, giving fruit peels can increase protein digestibility and utilization, which can be linked to increased protein content in broiler chicken meat. For the record, an increase in protein content in meat is a good indicator of meat’s chemical quality. In general, increasing the protein content of meat will improve its physical quality, particularly in terms of water-holding capacity and cooking loss from chicken meat. Concerning protein’s ability to bind water, Mir *et al*. [51] stated that increasing protein content in meat can increase moisture content and water-holding capacity and reduce cooking loss from meat. Nuriyasa *et al*. [52] found that feeding fermented banana peel meal to Indonesian native chickens reduced fat content in the meat. Ghosh *et al*. [41] discovered a hypolipidemic effect of the fruit peel when supplementing broiler chickens with pomegranate peel infusion. Given the hydrophobic nature of fat, this decrease in fat can explain the increase in water-holding capacity and decrease in cooking loss observed in the previous studies of broiler chickens fed fruit peel. Various active ingredients as antioxidant sources in fruit peel are likely to be responsible for improving antioxidant status and inhibiting fat peroxidation in broiler meat given fruit peel. Fruit peel antioxidants, particularly polyphenols, are responsible for inhibiting oxidative stress in broilers and fat peroxidation in broiler meat [41].

Aside from studies that show that using fruit peels improves growth performance, several other studies have produced conflicting results. Duwa *et al*. [53] reported that feeding banana peel meal to broiler chickens had a negative impact on their growth and physiological condition. Abel *et al*. [4] found similar results when feeding banana peel meals to broiler chickens. They found that broilers fed banana peel meal had lower growth and digestibility. Another study by Ghasemi-Sadabadi *et al*. [22] and Ghasemi-Sadabadi *et al*. [54] found that using 8% pomegranate peel in diets significantly impaired nutrient digestibility and decreased broiler growth rate. Another study, conducted by Vlaicu *et al*. [10], showed that grapefruit peel powder supplementation reduced the growth performance of broiler chickens when compared with controls. Furthermore, Alefzadeh *et al*. [55] found that administering dried orange peel powder at a level of 4 g/kg of feed had a negative impact on broiler chicken growth performance. In line with this, Aziz Ur Rahman *et al*. [56] reported that feeding potato peel powder (10%, 15%, and 20% of diets) had a negative effect on broiler growth performance at 4 weeks of age. This slowed growth rate corresponded to the birds’ impaired nutrient digestibility when fed potato peel powder. In this case, the chicks’ inability to break the non-starch polysaccharides (NSPs) present in potato peel powder appeared to be responsible for the broiler chickens’ impaired digestibility and, thus growth performance.

In fact, the exact reasons for the negative impact of using fruit peel on broiler chicken growth are unknown. However, the presence of several antinutrients and toxins in fruit peels may have a negative impact on digestibility, reducing broiler chicken growth rate [53]. Furthermore, Ghasemi-Sadabadi *et al*. [22] reported that tannins and toxins in fruit peels can affect the physiological condition of broiler chickens, as evidenced by a decrease in RBC, hemoglobin (Hb), and packed cell value (PCV). Furthermore, the presence of tannins and toxins in fruit peels may have a negative impact on the structure of the small intestine, resulting in lowered villus height in the duodenum, jejunum, and ileum and compromised nutrient absorption [54]. Similarly, Adekeye *et al*. [5] proposed that the high crude fiber content in fruit peels is very likely to be responsible for the decreased nutrient digestibility, which results in a decrease in broiler growth performance. Moreover, Vlaicu *et al*. [10] reported that fruit peel is very likely to affect the flavor of the feed, resulting in a decrease in broiler consumption. Alefzadeh *et al*. [55] found that administering dried orange (*C. sinensis*) peel powder affected the flavor and appetite of chickens, reducing feed consumption and, ultimately, broiler chicken growth.

## Effect of Fruit Peels on Health of Broilers

Health is an important factor in broiler chicken production. Healthy chickens will use more energy from feed for growth, whereas sick chickens will use most of their energy for recovery, causing their growth to slow. When the chicken becomes ill, medical expenses will also increase. Broiler producers have widely used phytochemicals to improve the health of broiler chickens after the ban on AGP in feed [6]. Fruit peels, in particular, are a rich source of phytochemicals in plant-based ingredients. As discussed in the previous section, fruit peels are rich in antimicrobial, antioxidant, anti-inflammatory, immunomodulatory components, and other components that are beneficial to the health of broiler chickens [12]. Several studies have been conducted to investigate the potential of various fruit peels as functional feeds and feed additives to improve broiler chicken health (Table-3) [10, 22–24, 37, 39, 42–46, 49, 54, 57, 58]. Various fruit peels have been shown to improve the population and diversity of microbes in the digestive tract [10, 42], immune system [37, 39, 43, 49], antioxidant status [23, 24], physiological conditions [39, 44], and the function of vital organs, such as the liver in broiler chickens [22].

**Table-3:** Effects of fruit peels on health of broilers.

Fruit peels	Active compounds	Levels in diets	Effects on broilers	References
Orange peel or grapefruit peel powder	Vitamin E, lutein and zeaxanthin, polyphenols and Zinc	2% of diets	Decreased numbers of *Enterobacteriaceae*, *Escherichia coli*, *Staphylococcus* spp. and increased *Lactobacillus* spp. in broiler small intestine	[10]
Pomegranate peel powder	Phenols, hydrolysable tannins and condensed tannins	4% and 8%	At 4% increased IgG, IgM and total immunoglobulin levels, and decreased meat MDA concentration	[22]
Pomegranate peel powder	Polyphenols, hydrolysable tannins and condensed tannins	4% and 8% of diets	At 4% reduced AST, ALT and MDA, while increasing TAC, SOD, GPx activities, IgG, IgM and total immunoglobulin	[22]
Pomegranate peel extract	Phenols, proanthocyanin, punicalagin, ellagic acid, oleuropein, gallic acid, caffeic acid, and catechin	2.5, 5.0, 7.5 and 10 g/kg	At 7.5 g/kg reduced hepatic lipogenesis through upregulation of PPRAα, increased antioxidant enzymes (CAT and SOD) and prevented lipid peroxidation	[23]
Banana peel powder	Phenols, gallocatechin, epigallocatechin, epigallocatechin gallate	0.5, 1.0 and 2.0%	At 0.5% increased serum SOD activity and lowered serum MDA levels, while the liver mRNA expression of Nrf2, SOD, CAT, GPx, and HO-1 were upregulated at 0.5% and 1%	[24]
Pomegranate peel extract	Phenolic compounds and flavonoids	0.05% and 0.10%	At 0.1% improved serum lysozyme activity and increased the relative weight of the bursa of Fabricius and thymus gland to live body weight of broiler	[37]
Prickly pear fruits peel powder	Phenols and flavonoid	5, 10 and 15% of diets	At all levels improved immunity, increased serum globulin and glucose levels, decreased serum triglyceride and cholesterol levels of broilers	[39]
Red dragon fruit peel meal	NM	0.5, 1.0 and 1.5%	At all levels decreased ileal coliform counts	[42]
Pomegranate peel powder	Phenols	2, 3 and 4 g/kg diets	Improved blood serum metabolites and immunological parameters of broilers	[43]
Pomegranate peel powder	Flavonoids, catechins, ellagic acid, flavones, and anthocyanidins	0.25, 0.50, 1.00, and 1.50%	Increased total protein, globulin, RBCs, Hb, PCV, and WBCs, while decreasing AST, and ALT compared to the control group	[44]
Pomegranate peel extract	Phenolic compounds, flavonoids, saponins and tannins	10 g/100 kg diet	Reduced total aerobic bacteria in broiler intestine	[45]
Sweet citrus peel powder	NM	2, 4, and 6 g/L of water	Decreased total bacteria counts in ileum of broilers	[46]
Dried sweet orange (*Citrus sinensis*) peel	Vitamin C and polyphenols	1.5% and 3.0%	Improved immune responses (total anti-SRBC titer, IgG and IgM levels) of broilers	[49]
Pomegranate peel powder	Hydrolysable tannins and condensed tannins	4% and 8% of diets	At 4% increased *Lactobacillus* population in the small intestine of broilers	[54]
*Citrus sinensis* peel powder	NM	1% and 2% of diets	Improved primary and secondary antibody responses to SRBC and against PHA-P antigen, and increased antibodies titer against Newcastle disease during the secondary antibody response	[57]
Sweet orange (*Citrus sinensis*) peel extract	NM	1000 and 1250ppm	At 1250 ppm increased antibody titer response to SRBC, IgG and IgM levels	[58]

ALT=Alanine transaminase, AST=Aspartate transferase, CAT=Catalase, GPx=Glutathione peroxidase, Hb=Hemoglobin, IgA=Immunoglobulin A, IgG=Immunoglobulin G, MDA=Malondialdehyde, NM=Not mentioned, PCV=Packed cell volume, PHA-P=Phytohemagglutinin-P, RBCs=Red blood cells, SOD=Superoxide dismutase, SRBC=Sheep red blood cells, TAC=Total antioxidant capacity, WBCs=White blood cells

Several active ingredients found in fruit peels have been associated with the ability of fruit peels to increase the population and diversity of microbes in broiler digestive tracts [10, 42]. Some antimicrobial compounds found in fruit peels are responsible for the increased microbial population and diversity in broiler intestines [12]. In this case, Vlaicu *et al*. [10] and Akintunde *et al*. [11] reported that several components in fruit peels, such as saponins, alkaloids, polyphenols, zinc, and magnesium, have antibacterial properties and can play an important role in improving the microbial ecosystem in broiler chicken digestive tracts. Several factors must be considered when using fruit peel as an antimicrobial agent in broiler chicken intestines. In general, the antimicrobial capacity of fruit peel varies depending on several factors, including the type of fruit peel, the extraction method (when used as an extract), the application to broiler chickens (as functional feed or feed additive), the delivery methods to broilers (via feed or drinking water), and the type and level of active ingredients contained in the fruit peel [30, 36]. Saleem and Saeed [30] compared the antimicrobial activity of three fruit peels, including yellow lemon, orange, and banana peels, in relation to the different types and levels of active ingredients contained in fruit peels and their relationship to the antimicrobial activity found in fruit peels. The latter researchers reported that yellow lemon, orange, and banana peels had the highest antimicrobial activity against Gram-positive and Gram-negative bacteria, microscopic filamentous fungi, and yeast. Saleem and Saeed [30] also demonstrated that the best fruit peel extraction processes used distilled water, methanol, ethanol, and ethyl acetate. Fruit peel is a part of the fruit that contains a fairly high fiber content. This fiber can often interfere with nutrient digestion in chickens, but it can also play an important role as a prebiotic, improving the conditions of the microbial ecosystem in the digestive tract, particularly the intestine in broiler chickens [36]. Fiber, particularly oligosaccharides, can promote the growth of endogenous beneficial bacteria (e.g., lactic acid bacteria), improve intestinal microbial balance, and boost chick immune defense. Sugiharto [6] has gone into great detail about how prebiotics improve the intestinal microbial ecosystem in broiler chickens.

Several studies have found a link between the condition of the intestinal ecosystem and the immune system in broiler chickens [6, 59]. In this case, giving fruit peels to broiler chickens have a positive impact not only on the condition of the microbial ecosystem in the gut but also on the development and immune response of broiler chickens [59]. Sugiharto [6] and Sugiharto and Ranjitkar [59] reviewed the details of the relationship between the condition of the microbial ecosystem in the gut and the immune status of broiler chickens. Anthocyanins are active components (a group of flavonoids) found in fruit peels that have the ability to modulate the immune system [60]. Based on these findings, the improvement of the immune system in chickens treated with fruit peel can be attributed to anthocyanins’ ability to stimulate the immune response and control inflammatory responses in broiler chickens [61]. In terms of antioxidant capacity, anthocyanins found in fruit peels are also very likely to be capable of preventing chicken immunological stress [60, 61].

Apart from their prebiotic role in broiler chicken intestines, oligosaccharides found in fruit peels may play an important role in improving broiler chicken immune systems. Csernus *et al*. [61] revealed that oligosaccharides can modulate immune responses and control inflammation in broiler chickens. Such conditions are expected to improve broiler chicken health. The presence of phenolic components in fruit peels is also often associated with improved immune system function in broiler chickens. According to the findings of Pourhossein *et al*. [49], the use of sweet orange (*C. sinensis*) peel can improve the immune response of broiler chickens. They further explained that, in addition to playing an important role in the body’s antioxidant system, flavonoids found in fruit peels can modulate and synergize with the immune system, improving chickens’ ability to defend themselves against incoming pathogens.

Fruit peels are also rich in hydrolyzable tannins and condensed tannins, which can help boost the immune system. Ghasemi-Sadabadi *et al*. [22] reported that the use of hydrolyzable tannins and condensed tannins found in pomegranate peel powder increased IgG, IgM, and total Ig levels in broiler chickens. In this case, hydrolyzable tannins and condensed tannins reduce oxidative stress, which benefits the integrity, development, and function of lymphoid tissues and organs in producing immune cells. These active components, which are abundant in fruit peels [11, 26], are reported to be able to reduce free radicals and oxidants to maintain the integrity and function of lymphoid tissues and organs in the production of immune cells and antibodies. In addition, vitamin C has been shown to stimulate the immune response in broiler chickens [57]. Regarding the fiber content of fruit peel, Abdulameer [57] proposed that the fiber in fruit peel could promote phagocytosis and increase the secretion of ILs and interferons, thereby improving the immune responses of broiler chickens.

Damage to lymphoid tissues and organs must be avoided because it has a negative impact on the production and maturation of immune cells and antibodies. Several studies have found that using fruit peel improves antioxidant status in broiler chickens. For example, Ahmadipour *et al*. [23] and Ghasemi-Sadabadi *et al*. [22] found that pomegranate peel extract increased total antioxidant capacity, antioxidant enzyme (CAT, SOD, and GPx) activity, and prevented lipid peroxidation. Chueh *et al*. [24] also found that giving banana peel meal increased serum SOD activity while decreasing serum MDA levels. Banana peel meal can also upregulate liver mRNA expression of nuclear factor E2-related factor 2, SOD, CAT, and GPx, which improves broiler chickens’ response to oxidative stress. Components of antioxidant sources found in fruit peels, such as phenols, proanthocyanins, punicalagin, ellagic acid, oleuropein, gallic acid, caffeic acid, catechins, and others, play an important role in maintaining antioxidant status in broiler chicken bodies. Antioxidant components will work to neutralize or reduce excessive reactive oxygen species in the body, preventing lipid peroxidation and damage to cells and tissues, particularly lymphoid cells and tissues in the chicken body [24]. Furthermore, Anantachoke *et al*. [62] used *in vitro* studies to explain how exogenous antioxidants can affect the production of antioxidant enzymes in the body’s cells. They confirmed that antioxidants in fruit peels can increase the mRNA and protein expressions of antioxidant enzymes, such as CAT, GPx-1, and manganese-SOD, thereby improving the body’s overall antioxidant status.

At a certain level, using fruit peel as an additive or feed ingredient can improve the physiological condition of broiler chickens. Elnaggar *et al*. [44] showed that using pomegranate peel powder in feed increased total protein, globulin, RBCs, Hb, PCV, and WBCs, while decreasing aspartate transferase and alanine transaminase compared with the control group. In general, improvements in blood cell profile and blood biochemistry are associated with improved physiological conditions and health in broiler chickens. Improvements in these blood parameters have also been shown to correlate with increases in growth rate and FCR in broiler chickens [44]. The role of the various active ingredients contained in the fruit peel was inextricably linked to the improvement in the physiological condition of the chickens given the fruit peel. Hu *et al*. [63] confirmed in a mouse study that giving citrus peel powder positively impacts health and liver function and can prevent dyslipidemia conditions. In this case, the high polyphenol content of fruit peels is important in protecting the liver from potential damage from oxidative stress in broiler chickens [44]. Furthermore, polyphenols in fruit peels can improve fat metabolism, preventing fat accumulation in the liver and dyslipidemia conditions in the chicken body [63]. According to Ahmadipour *et al*. [23], dietary supplementation with pomegranate peel extract reduced hepatic lipogenesis by upregulating peroxisome proliferator-activated receptor α (PPRAα). For the record, the liver is a vital organ in the body that performs various functions, such as protein synthesis, detoxification of toxic substances, production of cholesterol and hormones, and destruction of damaged RBCs. Based on the data presented above, improving liver function and condition will positively impact broiler chickens physiological condition. Concerning the effect of fruit peels on kidney health and function, several studies found no significant effect on uric acid and creatinine levels, which are indicators of kidney health and function in broiler chickens [24, 44]. Unlike the research on broiler chickens, Eldin *et al*. [64] found that Mandarin fruit peel hydroethanolic extract could protect liver and kidney health and function by enhancing the antioxidant defense system, anti-inflammatory activity, and antiapoptotic action in Wistar rats. On this basis, it is important to conduct further research on the effect of giving fruit peels on uric acid and creatinine levels.

Several studies reported different results than the ones mentioned above. Nurmeiliasari *et al*. [65] reported that there was no effect of red dragon (*Hylocereus polyrhizus*) fruit peel powder at levels of 2.43, 3.64, and 4.86 g/bird on the physiological conditions of broiler chickens as indicated by the blood profile. Similarly, Kumar *et al*. [66] found no effect of pomegranate (*Punica granatum*) peel extract (100-mg/kg diet) on broiler immune system. Ghasemi-Sadabadi *et al*. [22] reported that giving fruit peels had a negative impact on RBCs, Hb, and PCV in broilers by giving 8% pomegranate peel powder. When pomegranate peel was added to feed at 4%, the results were different. Indeed, 4% pomegranate peel has been shown to improve the physiological conditions and immunity of broiler chickens [22]. Based on these conditions, it can be assumed that the level of use in feed has a significant impact on determining the effect of fruit peel on the health of broiler chickens. The more fruit peel used in feed, the more antinutrients the chickens consume, which has a negative impact on nutrient utilization and the physiological condition of broiler chickens. Different types of fruit peels are also a factor that may influence the effectiveness of fruit peels in improving the health condition of chickens. This is due to the type and level of active components and antinutritional substances found in fruit peels. The fruit peel’s form, such as powder or extract, is also very likely to have an effect on its effectiveness in broiler chickens. In general, fruit peels in extract form contain more active ingredients than fruit peels in powder form, so fruit peels in extract form can be used in lower doses. However, no studies have been conducted that directly compare the efficacy of fruit peels in powder versus extract form. Fruit peel extract can be administered to broiler chickens via feed [37, 45] or drinking water [41, 46]. So far, no research has been conducted that examines the differences in the effects of the two delivery methods on broiler chickens in terms of whether there are differences in the effect of fruit peel due to different delivery methods.

## Optimizing the Use of Fruit Peels in Broiler Production

### Fermentation

Fermentation is a simple method that broiler farmers use to improve the nutritional and functional values of non-conventional feed ingredients [59]. In terms of fruit peel fermentation, Mandey *et al*. [67] used pineapple peels. They showed that fermentation with mixed cultures containing *Candida parapsilosis*, *Candida melinis*, *Hansenula supbeliculosa*, *Hansenula malanga*, *Aspergillus niger*, *Aspergillus oryzae*, and *Saccharomyces cerevisiae* increased the protein, fat, Ca, P, and energy content of pineapple peel while decreasing crude fiber content. Oluremi *et al*. [3] found that fermentation reduced the content of antinutritional factors, such as oxalate, saponin, and phytic acid in sweet orange peel. Furthermore, Ariana *et al*. [68] fed fermented dragon fruit skin to broilers and found that chicks fed fermented dragon fruit (*H. polyrhizus*) skin at levels as high as 5% and 7% in the feed had a higher final body weight than chickens fed control feed (without fermented dragon fruit skins). The latter authors explained that administering fermented dragon fruit skin can improve ecological conditions in broiler chicken digestive tracts, thereby improving digestion and nutrient absorption. In addition to increasing nutritional value, fermentation can increase the content of beneficial microbes, such as lactic acid bacteria, which have a positive impact on broiler chickens [59]. Sugiharto *et al*. [69] previously reported that fermentation with *Chrysonilia crassa* and *Bacillus subtilis* could reduce fiber content while increasing protein and energy values in banana peels. According to an *in vivo* study, inclusion of fermented banana peels up to 15% in the ration has no negative impact on the growth performance of broiler chickens. In this case, using fermented banana peel instead of maize as a conventional energy source for broiler chickens can reduce the use of maize. In line with this, Shihah *et al*. [70] reported that using fermented lime (*Citrus aurantifolia*) peels up to 3% in diets had no significant effect on the growth, feed intake, and FCR of broiler chickens. Indeed, the administration of fermented lime (*C. aurantifolia*) peels improved broiler immune competencies, as evidenced by a greater relative weight of the Fabricius bursa than the control.

Although many studies have found that fermentation improves the nutritional and functional composition of fruit peels, using fermented fruit peels for broiler chickens should be done with caution. This is because several studies have found fermented fruit peel to be ineffective as an alternative feed ingredient for broiler chickens. Oluremi *et al*. [71] used fermented sweet orange fruit peel in feed as an alternative energy source. However, they reported a negative effect of fermented sweet orange fruit peel on the growth of broiler chickens. Koni [72] found that feeding fermented banana peel to broiler chickens had a negative effect. The author stated that fermented banana had no negative impact in the feed at 5%, but at 10%, the growth rate of the chicken decreased as feed intake decreased. The high crude fiber content was very likely to reduce feed digestibility, resulting in lower consumption and growth rates in chickens. When fermenting fruit peels, the starter used, the duration of fermentation, and the fermentation conditions must be considered so that the fermented fruit peels produced have nutritional value in accordance with the needs of broiler chickens [71]. The proportion of fermented peel as a feed ingredient for broiler chickens must also be considered because there are still antinutritional components present, and the high crude fiber content can inhibit nutrient utilization by broiler chickens [67, 69].

### Extraction

Fruit peel can be extracted to obtain various active components for use as a feed additive for broiler chickens, in addition to being used as an alternative feed ingredient. Singh and Immanuel [73] extracted antioxidants from various fruit peels, including pomegranate, lemon, and orange. They reported that the antioxidants extracted from the three fruit peels differed depending on the type of fruit peel, with pomegranate peel as the main source of antioxidants, followed by lemon peel and orange peel. For reasons of hygiene and abundance, ethanol and water are the most commonly used solvents to extract the active components found in fruit peels [73].

Kishawy *et al*. [37] conducted a study that used fruit peel extract as a feed additive for broiler chickens. Pomegranate peel extract, which is rich in phenolic compounds and flavonoids, is used as a natural antioxidant source for broiler chickens. Natural antioxidants are very beneficial in reducing the use of synthetic antioxidant sources, which are believed to cause health problems in broiler consumers. Ahmadipour *et al*. [23] used fruit peel extract to improve physiological and immune competencies, and Hamady *et al*. [45] used it to improve the microbial population in the small intestine of broiler chickens.

In general, using fruit peels in the form of dried powder is believed to be more advantageous because it may reduce the proportion of conventional feed ingredients used as energy sources. However, because the extraction process can reduce the content of antinutrients in the fruit peel, the application of fruit peel in the extract form is believed to be more effective in impacting the physiological conditions and health of chickens. The extraction process can also increase the availability of the active components in the fruit peel, allowing the chickens to use it better.

### Distillation

Distillation is the most commonly used method for obtaining essential oils. Distillation separates chemicals based on differences in the speed or ease of evaporation (volatility) of the materials. The fruit peel powder is boiled to evaporate and then cooled back into liquid form during the distillation process. Several solvents, such as methanol, hexane, and distilled water, are often used in the distillation process [74]. Some factors have been reported to influence the amount of essential oil produced by fruit peel and its active compounds. Among these, factors are the type of fruit peel, the solvent used, the heating time, and the maturity stage of fruits [74, 75].

Several researchers have also reported using essential oils derived from fruit peels in broilers. Sahu *et al*. [76] reported that using essential oils from lemon and orange peel either alone or in combination in feed (200 mg/kg feed) during the summer can increase growth rate, gut health, and profit margins in broiler production. In another study, Erhan and Bölükbaşı [77] reported that giving 3 mL/kg of orange peel essential oil had a positive effect on the growth performance, jejunal microflora, and jejunal morphology of broiler chickens. Aydin and Alçiçek [78] showed that supplementing broiler chicken diets with essential oil extracted from orange peel (50-, 100-, and 150-mg/kg diets) increased final body weight and carcass yield linearly. In general, essential oil is a concentrated liquid that contains a mixture of volatile compounds, such as antibacterial, antioxidants, and other active ingredients that are beneficial to the health and growth of broiler chickens [76, 77]. Puvaća *et al*. [79] provide an in-depth review of the use of essential oil in broiler production and an explanation of how essential oils can improve broiler growth and health.

### Combined use of fruit peels and other active ingredients

The use of fruit peel as an alternative feed ingredient or functional feed ingredient in broiler chicken diets is often hindered by the fiber content, antinutrients, and toxins in the fruit peel. Combining fruit peels with other active ingredients, such as enzymes, are one method for increasing fruit peel utilization by broiler chickens. Enzymes are chemical compounds that are commonly added to poultry feed to improve digestibility. Alefzadeh *et al*. [55] combined dried orange peel powder with multienzymes and found that multienzymes could compensate for the negative impact of dried orange peel powder on the growth rate of broiler chickens. In this case, multienzymes are very likely to compensate for the decreased digestibility and nutrient utilization caused by the inclusion of fruit peels in the feed. According to Aziz Ur Rahman *et al*. [56], supplementation with a mixture of exogenous enzymes (xylanase, mannanase, protease, and cellulase) can compensate for the negative effects of giving potato peel powder on the digestibility and growth performance of broilers.

Probiotics are feed additives that have been shown to improve broiler chicken digestibility and nutrient utilization [6]. Based on the information presented above, Abdel Baset *et al*. [43] combined pomegranate peel powder (2–4-g pomegranate peel powder/kg diet) and probiotic *Bacillus toyonensis* (1-cm^3^ probiotic/kg diet) to maximize the benefits of pomegranate peel and achieve a synergistic effect in broilers. Unfortunately, Abdel Baset *et al*. [43] discovered no synergistic effect of pomegranate peel powder and *B. toyonensis* on the performance or health of broiler chickens. Based on these findings, it is important to consider the best possible combination of fruit peel and probiotics. This is due to the ability of various probiotic microbes to help in the breakdown of NSPs and the reduction of antinutritional components found in fruit peels. Until now, there has been no research on combining fruit peel and probiotics for broiler chickens.

## Conclusion

Fruit peel has the potential to be an alternative feed ingredient for broiler chickens due to its high-energy content. Fruit peels also contain antibacterial, antioxidant, and immunostimulant components, making them potentially useful as feed additives for broiler chickens in the post-antibiotic era. However, the use of fruit peels for broiler production is often limited due to their high crude fiber content and the presence of antinutritional and toxic components that can interfere with the digestion and physiological functions of broilers. Therefore, further studies are needed to determine the optimal and safe levels of use for these peels in broiler chickens.

## Authors’ Contributions

SS: Drafted, revised, and approved the final manuscript.
